# Forward-backward translation, content validity, face validity, construct validity, criterion validity, test-retest reliability, and internal consistency of a questionnaire on patient acceptance of orthodontic retainer

**DOI:** 10.1371/journal.pone.0314853

**Published:** 2025-01-03

**Authors:** Zhi Kuan Saw, Jonathan Jun Xian Yuen, Asma Ashari, Fatima Ibrahim Bahemia, Yun Xuan Low, Nik Mukhriz Nik Mustapha, May Nak Lau

**Affiliations:** 1 Department of Family Oral Health, Faculty of Dentistry, Universiti Kebangsaan Malaysia (UKM), Kuala Lumpur, Malaysia; 2 Department of Paediatric Dentistry and Orthodontics, Faculty of Dentistry, Universiti Malaya (UM), Kuala Lumpur, Malaysia; 3 Centre for Paediatric Dentistry and Orthodontic Studies, Faculty of Dentistry, Universiti Teknologi MARA (UiTM), Selangor, Malaysia; Universidade Federal de Pelotas, BRAZIL

## Abstract

This study aimed to assess the validity and reliability of a questionnaire on patient acceptance of orthodontic retainers. The original questionnaire was forward- and backward-translated, followed by four validity tests (content validity, face validity, construct validity, criterion validity) and two reliability tests (test-retest reliability, internal consistency). Content validity was assessed by nine orthodontists who appraised the questionnaire’s representativeness, relevance, clarity, and necessity. Face validity was established through semi-structured in-depth interviews with 35 English-literate participants currently wearing orthodontic retainers. Construct validity was established through Exploratory Factor Analysis (EFA). For criterion validity, 107 participants concurrently answered the questionnaire and the Retainer-modified Malaysian Oral Health Impact Profile questionnaire. Test-retest reliability was verified by 34 subjects who responded to the questionnaire again after a two-week interval. Six revised items passed the threshold value of 0.78 for Item-Content Validity Index and Content Validity Ratio and were revised based on findings from the face validity test. Principal Component Analysis of EFA extracted information on only one component, and all items were positively correlated with the component matrix. Spearman’s rho value (*rs* = 0.490 and *r*s = 0.416) indicated a moderate correlation between the two questionnaires for criterion validity. Intraclass Correlation Coefficient ranged from 0.687 to 0.913, indicating moderate to excellent test-retest reliability. Cronbach’s alpha ranged from 0.687 to 0.913 indicating that none of the questionnaire items showed unacceptable or poor internal consistency. The questionnaire on patient acceptance of orthodontic retainers has been validated and can be used in both clinical and research settings.

## Introduction

Retention is the phase of orthodontic treatment in which teeth are maintained in their corrected positions after active tooth movement [1]. During this stage, retainers are prescribed to minimize relapse, which is the tendency of teeth to return to their pretreatment positions [2–4].

Retainers can be fixed, removable, or a combination of both and the retainer choice depends on factors include pretreatment occlusion, treatment results and oral hygiene [4]. It was found that orthodontists favor removable retainers over their fixed counterparts [5]. The two most common removable retainers prescribed are the Hawley retainer and the vacuum-formed retainer (VFR) [5].

In most cases, patients are instructed to wear removable retainers at night indefinitely [6]. However, achieving consistent long-term compliance remains challenging [7]. Discomfort caused by retainer wear has a negatively impact on patient’s acceptance and compliance [8]. A comfortable retainer that is well-accepted by patients for long-term use is key to improve retention outcomes and satisfaction [9].

To objectively assess patient acceptance on orthodontic retainers, a valid and reliable instrument is needed. Saleh et al. [10] modified a questionnaire originally developed by Ngan et al. [11] by reducing the questionnaire items from 14 to 10. In their questionnaire, Saleh et al. used visual analogue scale (VAS) to assess patient acceptance of orthodontic retainer in teeth biting, fitting of the appliance, speech, appearance, oral hygiene, durability, gingival irritation, swallowing, self-confidence, and comfort [10]. However, this questionnaire lacks validity and reliability testing. Similarly, Mohd Tahir et al. developed the Retainer-modified Oral Health Impact Profile for Malaysian adult (Retainer-modified S-OHIP(M)), assessing functional limitation, functional pain, physiological discomfort, physical disability, psychological disability, social disability, and handicap [12]. This questionnaire has only undergone face validity testing.

While both instruments aim to measure patient acceptance of orthodontic retainers, they have limitations in comprehensive validity and reliability testing, highlighting the need for a more robust tool. The present study aims to validate the questionnaire on patient acceptance of orthodontic retainers and assess its reliability.

## Methods

### Subjects and methods

This cross-sectional study was approved by the ethics committee of the Faculty of Dentistry Medical Ethics Committee (FDMEC), Universiti Malaya (DF CD2106/0016 (U)). Subject recruitment was conducted between June 1, 2021, and December 1, 2021. Four validity tests (content validity, face validity, construct validity, and criterion validity) and two reliability tests (test-retest reliability and internal consistency) were conducted. The STROBE (Strengthening the Reporting of Observational Studies in Epidemiology) guidelines were followed to ensure rigorous and transparent reporting of this study [13].

### Setting and participants

The inclusion criteria include those who were able to read and understand English, were currently wearing an orthodontic retainer, and were medically fit and healthy. Exclusion criteria include the inability to read or understand English, not currently wearing an orthodontic retainer, and the presence of medical conditions. Subjects were conveniently sampled from patients at the Faculty of Dentistry, Universiti Malaya. Written consent was obtained prior to the study via Google Form, including written consent from parents or guardians for minors.

The sample sizes of participants for each validity and reliability test are presented in Table 1. The sample size for each test was determined independently, as each test has a different recommended sample size.

**Table 1:** Sample size of participants for each validity and reliability test.

Test	N
Content validity	9^a^
Face validity	35^b^
Criterion validity	107^c^
Construct validity	105^d^
Reliability	34^e^

^a^ According to Lawshe (1975)

^b^ According to Yusoff (2019)

^c^ According to Kang (2021)

^d^ According to Anthoine et al. (2014)

^e^ According to Bujang and Baharum (2017)

### Study instrument

A questionnaire assessing patient acceptance of orthodontic retainers, initially developed by Ngan et al. [11] and later modified by Saleh et al. [10], was utilized in this study. Subjects were asked to indicate their responses to 10 questionnaire items on a 100 mm visual analog scale (VAS), with the scale anchored by extremes: “very comfortable” at the left end, “very uncomfortable” at the right end and a neutral midpoint labeled “mildly comfortable”. The questionnaire items addressed ten aspects of retainer wear, including “teeth biting”, “appliance fit”, “speech”, “appearance”, “oral hygiene”, “durability”, “gingival irritation”, “swallowing”, “self-confidence”, and “comfort”. Their responses were then quantified using a ruler by measuring from left to right (in millimeters).

### Forward-backward translation

Forward translation from English to Malay was performed independently by two translators. The two sets of translations were critically appraised by an expert panel consisting of three orthodontists and one expert in questionnaire translation and validation from the dental public health specialty. Amendments were made accordingly.

Backward translation into English was done independently by two other translators, who are native English speakers, were fluent in Malay, and were blinded to the original version. The two sets of translations were also critically appraised by an expert panel.

The final back-translated questionnaire was used for the subsequent content validity test.

### Content validity

Content validity was assessed by nine orthodontists from Universiti Malaya (UM), Universiti Kebangsaan Malaysia (UKM), and Universiti Teknologi Mara (UiTM) with at least ten years of experience. This panel size adheres the recommendation to have an odd number of experts with at least three members [14]. The questionnaire was evaluated in terms of representativeness, relevance, clarity, and necessity [14].

Representativeness, relevance, and clarity were assessed using a 4-point ordinal scale (Table 1) and analyzed using Item-Content Validity Index (I-CVI). The I-CVI was calculated as the number of experts giving a rating of 3 or 4 divided by the number of experts. An I-CVI ≥ 0.78 was considered acceptable [15]. Necessity was assessed using a 3-point ordinal scale (Table 2) and analyzed using Content Validity Ratio (CVR). CVR was calculated using the formula “CVR = (Ne—N/2)/(N/2)”, where N is the total number of experts and Ne is the number of experts indicating that the item is essential (with a rating of 3). The minimum acceptable CVR for nine panelists was 0.78 [14]. The experts were also asked to comment on the grammar and order of the wording.

**Table 2:** Scale for representativeness, relevancy, clarity, and necessity of questionnaire items.

Representativeness	Relevancy	Clarity	Necessity
1- Not representative 2- Item need some revision 3- Representative but need minor revision 4- Very representative	1- Not relevant 2- Item need some revision 3- Relevant but need minor revision 4- Very relevant	1- Not clear 2- Item need some revision 3- Clear but need minor revision 4- Very clear	1- Not necessary 2- Useful but not essential 3- Essential

The data were critically reviewed by an expert panel of three orthodontists with at least three years of experience from UM, UKM, and UiTM. Based on the results, two questionnaire items were rephrased, and the rephrased items were appraised by the nine orthodontists. The revised questionnaire was used for subsequent face validity tests.

### Face validity

**Fig 1:**
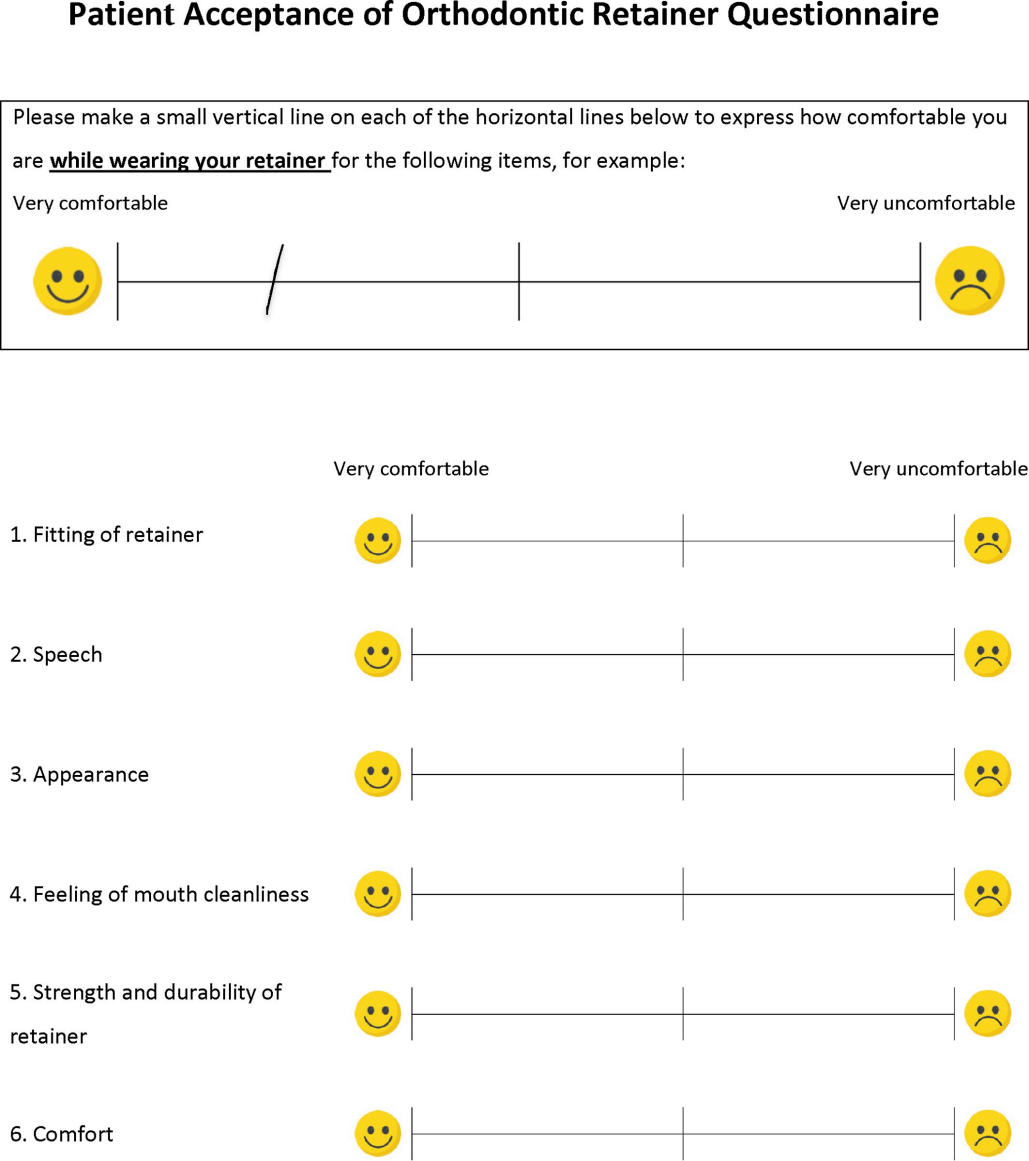
A sample of the questionnaire on patient acceptance of orthodontic retainers. The scale is 10 cm in length. The extent of patient discomfort is measured from the left end of the scale to the vertical line placed by the patient.

Pilot testing and interviewer training were conducted prior to the actual face validity testing. Sample recruitment continued until data saturation was achieved or a minimum sample size of 30 subjects was reached, based on the guidelines provided by Yusoff [16]. Subjects were asked to answer the questionnaire via Google Form, and then took part in a one-on-one semi structured in-depth interview on their comprehension and interpretation of each questionnaire item. These interviews were conducted via Google Meet and were recorded. The recorded audio was transcribed and thematically analyzed [17]. The expert panel then critically reviewed the findings, emphasizing difficulties encountered and suggestions from subjects. Based on this feedback, a final version of the questionnaire (Fig 1) was developed and later subjected to validity and reliability testing.

### Construct validity

A total of 105 participants were recruited for construct validity, meeting the minimum sample size of 100 recommended by Anthoine et al [18]. The subjects answered the questionnaire via Google Forms.

The suitability of the data for factor analysis was tested using the Kaiser–Meyer–Olkin (KMO) test, with KMO value of more than 0.50 indicating suitability for factor analysis [19]. On the other hand, Bartlett’s Test of Sphericity assessed whether the original matrix was an identity matrix, a value of less than 0.05 indicates the significance of data and was therefore acceptable for factor analysis.

Principal Components Analysis (PCA) was used to extract the maximum variance from the dataset for each component. Total Variance Explained shows how the data are spread (variance) among the six questionnaire items. According to the Kaiser criterion, components with eigenvalues greater than 1 should be retained—as it accounts for more variance than a single item, justifying the combination of those items into a factor [20]. However, this holds true only if each item contributes one unit of variance. Pett et al. advised that the Kaiser Criterion should only be used in PCA when the total variance is accounted for during extraction [21].

The Component Matrix determines the correlation (also known as factor loadings) between the component and the variables. The values can range from -1 to 1, with values near -1 or 1 indicating strong correlations, and values near 0 indicating weak correlations. A cutoff of 0.5 was applied as such loadings were considered significant [22].

### Criterion validity

A total of 107 subjects were recruited for criterion validity. Based on power calculations, a sample size of 92 was required to detect a medium-sized correlation (effect size = 0.3) between the two questionnaires, with 90% power [23, 24]. Additionally, it met the minimum requirement of 30 as recommended by Roscoe [25]. Subjects were asked to answer the current questionnaire and the Retainer-modified S-OHIP(M) concurrently [26].

The Retainer-modified S-OHIP (M) questionnaire uses the following scale and scoring system: ‘Very often’, ‘Quite often’, ‘Sometimes’, ‘Seldom’, and ‘Never’, which are assigned scores of 4, 3, 2, 1, and 0, respectively. Data with more than three “Don’t Know” responses were excluded, while for data with one or two “Don’t Know” responses, the mean score for that item was imputed [26]. The Retainer-modified S-OHIP(M) questionnaire uses two scoring methods, the additive score (ADD) and simple count (SC). ADD was counted as the sum of the scores of all 14 items, whereas SC was the count of the number of questionnaire items rated as ‘Very often’ and ‘Quite often’. The ranges of the scores for the ADD and SC were 0 to 56 and 0 to 14, respectively [25]. The data were analyzed for normality followed by Spearman Correlation Coefficient analysis.

### Reliability

The questionnaire was self-administered by 34 subjects twice, two weeks apart, for test-retest reliability and internal consistency tests. According to Bujang and Baharum, a minimum sample size of 30 is required to detect a value of 0.50 for the Intraclass Correlation Coefficient (ICC), with an alpha of 0.05 and a power of 90% [27]. The sample size was inflated to 34 to include a 10% drop-out rate. A two-week interval was used to minimize “carry-over” or memory effects and potential changes in retainers’ or patients’ conditions [28]. Test-retest reliability was assessed by calculating the ICC from the repeated administration of the questionnaire with values less than 0.5 indicating poor reliability, 0.5 to less than 0.75 indicating moderate reliability, 0.75 to less than 0.9 indicating good reliability, and 0.90 to 1.00 indicating excellent reliability [29]. Internal consistency was measured using Cronbach’s Alpha and interpreted as excellent (> 0.9), good (> 0.8), acceptable (> 0.7), questionable (> 0.6), poor (> 0.5), and unacceptable (< 0.5) [30].

### Statistical analysis

Statistical analyses were performed using the IBM Statistical Package for Social Sciences (SPSS) statistical analysis software Version 26.0 for Windows (IBM Corporation, NY). The collected data were treated as numerical data. The Shapiro‒Wilk test was used to assess the normality of the data distribution. Means and standard deviations were calculated for parametric data, while medians and 1st and 3rd quartiles were calculated for nonparametric data. The I-CVI and CVR were calculated for content validity. For criterion validity, a nonparametric Spearman Correlation Coefficient was calculated, as the ADD and SC scores of the Retainer-modified S-OHIP(M) were not normally distributed and had highly positively skewed data distributions. For reliability, a two-way mixed effects model was used to generate an ICC with 95% CI for continuous variables to assess test-retest reliability, and Cronbach’s Alpha was used to measure internal consistency.

## Results

### Content validity

Table 3 shows the content validity results. Based on expert feedback, the terms “tooth biting”, “gingival irritation”, “swallowing”, and “self-confidence” were discarded because they failed to meet the minimum acceptable CVR value of 0.78 for necessity [14].

**Table 3:** Content validity results.

	I-CVI (Representativeness)	I-CVI (Relevance)	I-CVI (Clarity)	CVR
Teeth Biting	1.00	1.00	0.89	0.56^⟊^
Fitting of Appliance	1.00	1.00	1.00	1.00
Speech	1.00	0.89	0.89	1.00
Appearance	0.89	0.89	0.89	0.78
Oral Hygiene	0.78	0.89	0.78	0.56^⟊^
Durability	0.89	1.00	1.00	0.33^⟊^
Gingival Irritation	1.00	1.00	0.78	0.56^⟊^
Swallowing	1.00	1.00	1.00	0.33^⟊^
Self Confidence	0.78	0.78	0.78	0.33^⟊^
Comfort	1.00	1.00	1.00	1.00

The experts expressed concerns that they were uncertain of “oral hygiene” and “durability” constructs. These items were revised to “cleanliness of mouth” and “robustness of retainer”, respectively. As shown in Table 4, the revised items scored above the minimum acceptable CVR values for necessity when they were re-evaluated for content validity.

**Table 4:** Content validity results for revised questionnaire items.

	I-CVI (Representativeness)	I-CVI (Relevance)	I-CVI (Clarity)	CVR
**Mouth Cleanliness**	0.89	1.0	0.78	0.78
**Robustness of Retainer**	0.89	1.0	0.78	0.78

I-CVI: Item-Content Validity Index; CVR: Content Validity Ratio.

### Face validity

A total of 35 respondents, with a mean age of 23.03 years and a standard deviation of 3.74 years, were interviewed for face validity. Table 5 shows the subjects’ demographic data, and S1 Table shows the thematic analysis data and verbatim consent for face validity.

**Table 5:** Subject demographics for face validity, n = 35.

Category	n	Percentage (%)
**Gender**		
Male	8	22.9
Female	27	77.1
**Ethnicity**		
Malay	12	34.3
Indian	2	5.7
Chinese	20	57.1
Others	1	2.9
**Age (years)**		
0–14 (Young age)	0	0
15–64 (Working age)	35	100
> 64 (Old age)	0	0
**Highest academic level**		
None	0	0
Primary School	0	0
Secondary School	0	0
Higher Education	35	100

The two items that caused confusion were “mouth cleanliness” and “robustness of retainer”. The expert panel decided to rephrase the items to “feeling of cleanliness of mouth” and “strength and durability of retainer”, respectively.

### Construct validity

Exploratory Factor Analysis was applied to determine the factor structure among the 6 items related to patient acceptance of orthodontic retainers (Table 6). The KMO measure of sampling adequacy was 0.765, above the suggested value of 0.5, and Bartlett’s test of sphericity was significant, which is < 0.001 (with Χ2 = 177.545, Df = 15). The data were deemed suitable for Principal Component Analysis for construct validity. In the present study, all initial communalities were above the threshold of 0.3 [31], and all loading factors were above 0.5. The EFA results for all 6 items revealed only one component with eigenvalues greater than 1. The results showed that the component explained 49.76% of the variance.

**Table 6:** Exploratory factor analysis.

Items	Factor loading
Fitting of retainer	0.768
Speech	0.683
Appearance	0.741
Feeling of mouth cleanliness	0.622
Strength and durability of retainer	0.647
Comfort	0.758
**Eigenvalues**	2.986
**% of variance**	49.760

Extraction method: Principal Component Analysis

### Criterion validity

The Shapiro‒Wilk test showed that the p value of the mean VAS score for the current questionnaire was more than 0.05 (p = 0.087), indicating normally distributed data. However, the p values of the ADD and SC score for the Retainer-modified S-OHIP(M) were less than 0.05 (p = 0.005 and p < 0.001, respectively), indicating that the data were not normally distributed. The Spearman Correlation Coefficient test was selected for criterion validity testing.

The mean and standard deviation of the average VAS score for the current questionnaire were 3.56 and 1.51, respectively. The median and interquartile range for the ADD of Retainer-modified S-OHIP(M) were 12.00 (minimum 0; maximum 33) and 10.00, respectively. The median and interquartile range for SC of Retainer-modified S-OHIP(M) were 1.00 (minimum 0; maximum 7) and 2.00, respectively.

The Spearman Correlation Coefficient test showed a significant correlation between the average VAS score and the ADD (rs = 0.490, n = 105, p < 0.01). The correlation between the average VAS score and SC score was also significant (rs = 0.416, n = 105, p <0.01). Both Spearman’s rho values (rs = 0.490 and rs = 0.416) indicated a moderate correlation between the two questionnaires.

### Reliability

A total of 34 subjects were recruited for reliability tests, with a mean age of 23.03 years and standard deviation of 3.80 years. All the questionnaire items exhibited moderate to excellent test-retest reliability, with ICC values ranging from 0.687 to 0.913. Cronbach’s alpha also ranged from 0.687 to 0.913, indicating that none of the questionnaire items showed unacceptable or poor internal consistency (Table 7).

**Table 7:** Test-retest reliability and internal consistency results.

Questionnaire Item	α	M	SD	ICC	95% CI	*P* Value
Fitting of Retainer	0.813	5.450	3.3399	0.813	0.627–0.907	< 0.001
Speech	0.861	7.862	4.0992	0.861	0.722–0.931	< 0.001
Appearance	0.687	4.709	3.1373	0.687	0.373–0.844	0.001
Feeling of Mouth Cleanliness	0.847	7.085	3.6794	0.847	0.693–0.923	< 0.001
Strength and Durability of Retainer	0.913	5.444	3.3657	0.913	0.826–0.957	< 0.001
Comfort	0.871	7.471	3.9779	0.871	0.742–0.936	< 0.001

α: Cronbach’s Alpha; M: Mean; SD: Standard Deviation

ICC: Intraclass Correlation Coefficient; 95% CI: 95% Confidence Interval.

## Discussion

The present study validates the Patient Acceptance of Orthodontic Retainer Questionnaire, a self-report instrument that evaluates patients’ perception of their orthodontic retainer while wearing it. The questionnaire utilizes a Visual Analog Scale (VAS) for data presentation and demonstrates good validity and reliability.

Questionnaires are among the most common methodologic approaches used in research studies [32]. Valid and reliable instruments are paramount to collecting accurate data, which is the foundation for generating meaningful and impactful results [33, 34]. Currently, validated questionnaires exist to assess patient satisfaction with dentures [35], implant-supported prostheses [36], and active orthodontic treatment [37].

To date, there is no validated questionnaire that assesses patient acceptance and satisfaction with orthodontic retainers although prior studies have utilized patient questionnaires on orthodontic retainers. [10, 12, 38]. Hichens et al. piloted a patient satisfaction questionnaire with good reliability, but it remains unvalidated [38]. The Retainer-modified OHIP(M) questionnaire by Mohd Tahir et al. have only undergone face validity testing [12]. Similarly, questionnaire by Saleh et al. on patient acceptance of retainers lacked validation as well [10]. This research addresses the critical gap by presenting the first validated questionnaire on patient acceptance of orthodontic retainers.

The questionnaire in the present study was modified from that developed by Saleh et al., consisting of ten items related to acceptability of orthodontic retainers [10]. During content validation, the expert panel identified “tooth biting,” “gingival irritation,” “swallowing,” and “self-confidence” as less relevant. The exclusion of "tooth biting" and "swallowing" likely reflects their limited relevance as these issues are typically transient [39]. Similarly, "gingival irritation" can usually be resolved effectively through clinical adjustments. The removal of “self-confidence” may reflect that it is a complex and subjective outcome influenced by multiple factors beyond the retainer alone [40], making it less suitable for inclusion in a tool focused on the retainer’s direct effects on patient acceptance. Of the six retained items, “speech,” “appearance” and “comfort” remained unchanged. “Oral hygiene” and “durability” were modified to “cleanliness of mouth” and “robustness of retainer” respectively, to make it more understandable to laypeople. The content validated questionnaire was deemed relevant and representative of the construct of patient’s perception towards retainers [41].

Face validity ensures the intended audience finds the questionnaire items understandable, relevant, and easy to answer [42]. During structured interviews, participants highlighted that the phrases “mouth cleanliness” and “robustness of retainer” were confusing. Many were uncertain whether “mouth cleanliness” referred to the cleanliness of retainer or how easy it was to clean. Similarly, over half of the participants found “robustness of retainer” vague, unsure whether it referred to the thickness, quality, size, or condition of the retainer. In response to the feedback, the expert panel revised these items to “feeling of cleanliness of mouth” and “strength and durability of retainer” for clarity. The item “fitting of the appliance” was changed to “fitting of the retainer” to enhance specificity.

The moderate correlation between the present questionnaire and the Retainer-modified S-OHIP(M) suggests both instruments measure related constructs of patient acceptance of orthodontic retainers. However, the moderate strength of this correlation also suggests that the two instruments are not entirely equivalent, which is expected given the differences in scope. The Retainer-modified S-OHIP(M) [12] was adapted from short version of the Malaysian OHIP-14 [26] that primarily assesses a broader range of impacts of oral disorders on quality of life. In contrast, the present questionnaire focuses more specifically on patient acceptance and comfort with orthodontic retainers. Thus, the present questionnaire potentially captures a more targeted insights into specific aspects of retainer acceptance which might not be fully captured by the Retainer-modified S-OHIP(M).

The questionnaire has undergone a rigorous validation process and has demonstrated moderate to excellent test-retest reliability, with ICC values of 0.69 to 0.91. In comparison, a study evaluating a questionnaire about complete dentures in edentulous patients reported an ICC of 0.37–0.83 [43]. Another study on the validation of a Korean version of the oral health impact profile questionnaire had an ICC of 0.40–0.61 [44]. The lower ICC in these studies could be attributed to the longer test-retest interval of two to three months, compared to two weeks in the present study. While the test-retest interval may vary depending on the construct to be measured, a period of approximately 2 weeks is typically regarded as appropriate [45].

Tests for internal consistency revealed good to excellent internal consistency for five items, with Cronbach’s Alpha ranging from 0.81 to 0.91, while "appearance" showed a questionable value of 0.69. Unlike the other five items which are more closely related to functional aspects, “appearance” pertains to a highly subjective aesthetic component [46]. Subjects might consistently prioritize these functional qualities over aesthetics, leading to strong internal consistency among these items. Furthermore, the perception of appearance may not always align with functional priorities, as some subjects may place less emphasis on appearance [47] and focus more on how the retainer feels or functions. This misalignment could weaken the relationship between appearance with the other items, leading to a lower Cronbach’s Alpha value.

As patient compliance toward orthodontic retainers heavily depends on their acceptance of the retainer, this validated instrument has the potential to predict future compliance [8]. It may reveal retainer overlooked design flaws that potentially reduce patient acceptance such as discomfort, speech, and esthetics embarrassment [48]. Along this vein, this validated instrument empowers clinicians to deliver patient-specific recommendations and address potential compliance issues, ensuring long-term compliance to retention regimes.

Furthermore, it serves as an effective tool for comparing patient acceptance across different retainer types. The questionnaire has been utilized in a randomized-controlled trial to assess subjects’ perceptions of the traditional Hawley retainer versus a modified version of the VFR after orthodontic expansion treatment [49]. Optimizing patient comfort improves compliance, which in turn lead to reduced occurrence of relapse, saving time, cost, and manpower [50].

A limitation of the present study is that all the participants were drawn from patients attending a single university clinic and may not fully represent the Malaysian population. Therefore, further validation in a broader population may be necessary to ensure generalizability. However, none of the questionnaire items were socially or ethnically specific. Another potential limitation is that the VAS may pose challenges for patients unfamiliar with this format, potentially influencing their responses. Future research should compare responses obtained via VAS with those from other instruments, such as Likert scales. Furthermore, long-term follow-up studies are recommended to assess how patient perceptions evolve over time and how these perceptions relate to actual compliance with retainer use.

## Conclusions

The ten-item questionnaire on patient acceptance of orthodontic retainers has been validated for content, face, construct, and criterion validity. Following the content validity test, four items were discarded, and three items were revised and rephrased. The final six-item questionnaire showed a moderate correlation with the relevant questionnaire, the Retainer-Modified S-OHIP(M). It also demonstrated moderate to excellent test-retest reliability, with none of the questionnaire items showing unacceptable or poor internal consistency. This validated questionnaire provides a reliable tool for assessing patient acceptance of orthodontic retainers and can be used effectively in clinical settings to gauge patient experiences and preferences.

## Supporting information

S1 Table Thematic analysis data and verbatim for face validity. (DOCX)
