# Supplementary material for: Forward-backward translation, content validity, face validity, construct validity, criterion validity, test-retest reliability, and internal consistency of a questionnaire on patient acceptance of orthodontic retainer
Source: PLoS One. 2025 Jan 3;20(1):e0314853. doi: 10.1371/journal.pone.0314853 (PMC11698520; doi:10.1371/journal.pone.0314853)
Supplement: S1 Table — (DOCX) [file pone.0314853.s001.docx]

**S1 Table: Thematic Analysis Data and Verbatim for Face Validity**

| **Theme** | **Sub-theme** | **Verbatim** | **[Decision]Action** |
| --- | --- | --- | --- |
| Mouth cleanliness | 1. Understood it as retainer cleanliness | **7 out of 35 subjects (20.0%)**  1) Every morning when I remove my retainers I will realise that there will be some whitish form of mucus, I think that is because of the saliva and over the night they are deposited there. (Subject 1, 41 years old Chinese female wearing upper Hawley retainer and lower VFR)  2) I chose the answer based on how I feel about my retainer. My retainer got a bit stained, I just don't feel very clean whenever I wear my retainer because the retainer itself I feel it is dirty. (Subject 2, 23 years old Chinese female wearing upper and lower VFR)  3) I will make sure my retainer is very clean, I brush my retainer almost like every single morning,when I wake up I will clean it and I will brush it with the toothpaste and then that's how I clean my retainer so I feel comfortable. (Subject 3, 20 years old Chinese female wearing upper and lower VFR)  4) Even I do my routine cleaning for my retainer, it still having like some calculus on my retainer.  I think for the mouth cleanliness can change to other words maybe how you clean your retainer. (Subject 4, 22 years old Chinese female wearing upper VFR)  5) So far no problem for me when wearing the retainer, I just like clean it every night, so I think when I put it in my mouth, it is clean, not causing any trouble. (Subject 5, 20 years old Chinese female wearing upper and lower VFR)  6)) As long as I don't eat or drink anything with it, it is fine. I think it is how clean the retainer is when I use it. I only use it at night right, I feel like it’s OK because I brush my teeth every time before sleeping, so when I use it, it looks clean. (Subject 6, 21 years old Indian female wearing upper and lower VFR)  7) I think mouth cleanliness is a bit confusing because the survey is regarding the retainer and then I only think to the aspect of retainer. Every morning when I take out the retainer, the retainer feels like a little bit sticky. I think it will cause some odour and some smell in my mouth, so I will usually wash it with shampoo. I am referring to the cleanliness of the retainer because it causes my mouth got the sticky stains after wearing it for one night. (Subject 7, 20 years old Chinese female wearing upper and lower VFR) | [Modified]  Changed to Feeling of mouth cleanliness |
|  | 2. Understood it as how easy or hard it is to clean the retainer | **6 out of 35 subjects (17.1%)**  1) My retainer has pores because I keep brushing it with toothpaste and toothbrush, so I have to keep it clean everyday. (Subject 8, 22 years old Malay female wearing upper Hawley retainer and lower bonded retainer)  2) It is very easy to clean, I also use Polident, for now I only wear it at night time, in the morning I soak it in the tablet for a few minutes. Does it mean how easy for me to clean my mouth when using the retainer? I am guessing it is about retainers because I always brush my teeth.  I think instead of asking about mouth cleanliness, maybe you can ask about the cleanliness of the retainer itself because I don't think people will change the way they brush their teeth just because they wear retainers right, because they will take off the retainers during brushing time. Wearing retainers doesn't affect my oral hygiene care routine at all, so I thought it was about retainers. When Drs issue retainers, usually they will teach you how to clean the retainers right, they won't teach you how to brush your teeth, so I thought maybe this one is for retainers. (Subject 9, 24 years old Malay male wearing upper and lower VFR)  3) It is just like how easy the retainers were to clean. I have to make sure it is clean, if not I will be uncomfortable, I have to like every morning and night I have to go to the toilet and clean it. (Subject 10, 19 years old Malay male wearing upper and lower VFR)  4) Means when I wear it, is it very easy to clean my retainers or is it very easy to make my teeth very sensitive. I think that the retainer is very hard to clean it, seems like everyday I just wash it manually I feel like it is not really clean. (Subject 11, 25 years old Chinese female wearing upper and lower VFR)  5) I have to clean the retainer often, I don't like to clean the retainer compared to when I was wearing braces, I have to use Polident, and need extra work. (Subject 12, 23 years old Malay female wearing upper Hawley retainer and lower VFR)  6) Sebelum atau selepas saya pakai, saya akan gosok gigi, kalau makan saya akan buka, dia senang untuk cuci gut, tak ade lah sampai tersangkut makanan dekat dawai tu. (Subject 13, 26 years old Malay female wearing upper and lower VFR and bonded retainer) | [Modified]  Changed to Feeling of mouth cleanliness |
|  | 3. Understood it as how easy or hard it is to clean both retainers and mouth | **1 out of 35 subjects (2.9%)**  1) I have to make sure my teeth is clean only I can wear the retainers, so I will need to spend more time in cleaning my teeth and also retainer, for the retainer I'm using Polident to clean it, and for my teeth I have to floss every single teeth and make sure there is no food or residue remaining then only I will wear it. My molar has grown to the very back end there, so I’ve to really clean the back there in order to ensure no food is stuck inside. (Subject 1, 41 years old Chinese female wearing upper Hawley retainer and lower VFR) | [Modified]  Changed to Feeling of mouth cleanliness |
|  | 4. Understood it as retainer cleanliness and how easy or hard it is to clean the retainer | **1 out of 35 subjects (2.9%)**  1) Because Hawley retainer is easier to remove when I eat and all that, that's why I prefer it better and it doesn't stain just like the clear retainer. I once broke my retainer, but I asked for the same type as well because it is easy to clean. (Subject 14, 23 years old Iban female wearing upper and lower Hawley retainers) | [Modified]  Changed to Feeling of mouth cleanliness |
|  | 5. Suggest ‘oral cleanliness’ to replace mouth cleanliness | **1 out of 35 subjects (2.9%)**  1) If you want to be more professional maybe you can change to oral cleanliness. (Subject 1, 33 years old Chinese female wearing upper Hawley retainer and lower VFR) | [Modified]  Changed to Feeling of mouth cleanliness |
|  | 6. Suggest ‘oral hygiene’ to replace mouth cleanliness | **2 out of 35 subjects (5.7%)**  1) Maybe can change the term to oral hygiene practice after wearing retainers. (Subject 4, 22 years old Chinese female wearing upper VFR)  2) I think instead of cleanliness you can use oral hygiene, just fits more professionally and in a big picture. (Subject 15, 22 years old Chinese female wearing upper and lower VFR) | [Dismissed] |
| Robustness of retainer | 1. Do not know the word ‘robustness’ | **1 out of 35 subjects (2.9%)**  1) Can you explain what robustness is, tak faham langsung. (Subject 16, 27 years old Malay female wearing upper and lower VFR) | [Modified]  Changed to Strength and durability of retainer |
|  | 2. Understood it as thickness of the retainer | **5 out of 35 subjects (14.3%)**  1) Because it is acrylic, it is difficult to keep my tongue like very comfortable, sometimes I keep biting on my tongue. ‘Fingers showing very thick’ (Subject 8, 22 years old Malay female wearing upper Hawley retainer and lower bonded retainer)  2) It is quite uncomfortable because I cannot bite properly and then because there is something in my mouth, before that the bite plane is very high, it was trimmed, but now is very thin already, but I still feel is quite high, even if I wear without a bite plane I think my front will also no occlude, so I hope that my retainer can be more thinner. (Subject 17, 23 years old Chinese male wearing upper modified Hawley retainer and lower VFR)  3) I mean the thickness of the retainer, it is not easily break, if it is enough thick, it won't break down. (Subject 5, 20 years old Chinese female wearing upper and lower VFR)  4) I think it's how thick the shape of it, the thickness, the shape and mould of it. (Subject 15, 22 years old Chinese female wearing upper and lower VFR)  5) I feel like the retainer is not that thick, it’s easy for me to talk with, and then it's not easy to break anyway unless I use a very large force which is not that possible anyway. My understanding of robustness will be like the thickness of the retainer in my mouth and if it is too big or too small or too wide. (Subject 14, 23 years old Iban female wearing upper and lower Hawley retainers) | [Modified]  Changed to Strength and durability of retainer |
|  | 3. Understood it as comfortability of the retainer | **1 out of 35 subjects (2.9%)**  1) When I first got the retainer, it was comfortable, but I think since it has been years, so it starts to become less comfortable. I took it as comfortability of retainer. (Subject 12, 23 years old Malay female wearing upper Hawley retainer and lower VFR) | [Modified]  Changed to Strength and durability of retainer |
|  | 4. Understood it as not easily fractured/ damaged/ break | **3 out of 35 subjects (8.6%)**  1) It is not easily fractured or falls down, I know it won't be damaged. (Subject 9, 24 years old Malay male wearing upper and lower VFR)  2) I mean the thickness of the retainer, it is not easily break, if it is enough thick, it won't break down. (Subject 5, 20 years old Chinese female wearing upper and lower VFR)  3) I feel like the retainer is not that thick, it’s easy for me to talk with, and then it's not easy to break anyway unless I use a very large force which is not that possible anyway. My understanding of robustness will be like the thickness of the retainer in my mouth and if it is too big or too small or too wide. (Subject 14, 23 years old Iban female wearing upper and lower Hawley retainers) | [Modified]  Changed to Strength and durability of retainer |
|  | 5. Understood it as quality of retainer/good condition | **3 out of 35 subjects (8.6%)**  1) I think the whole quality is quite good, for now it’s still in good condition. Because the VFR doesn't have any bubbles, I think for me it is quite good. I think robustness is about quality or condition of the retainer, I think also for me is the accuracy of the placement. (Subject 18, 25 years old Chinese female wearing upper and lower VFR)  2) I think it is the quality, the quality for my retainer is ok. (Subject 19, 22 years old Chinese male wearing upper and lower VFR)  3) Robustness means quality right? Because it is made of plastic and transparent. -after google search. (Subject 20, 24 years old Malay male wearing upper and lower VFR) | [Modified]  Changed to Strength and durability of retainer |
|  | 6. Understood it as the size and fitting of the retainer | **1 out of 35 subjects (2.9%)**  1) How the size of it? It actually fits and the size is just right. (Subject 6, 21 years old Indian female wearing upper and lower VFR) | [Modified]  Changed to Strength and durability of retainer |
|  | 7. Understood it as the shape and fitting of the retainer | **1 out of 35 subjects (2.9%)**  1) Is it supposed to be the shape of the retainer, condition of the retainer whether di cepat cabut ke tak. (Subject 21, 20 years old Malay female wearing upper and lower VFR) | [Modified]  Changed to Strength and durability of retainer |
|  | 8. Understood it as rigidity | **1 out of 35 subjects (2.9%)**  1) I think for me it is very comfortable because I think it is quite rigid, because I am not quite understand the meaning of robustness, I think is does it mean like rigidity of retainer. (Subject 22, 23 years old Chinese female wearing upper and lower Hawley retainers) | [Modified]  Changed to Strength and durability of retainer |
|  | 8. Understood it as accurate placement | **2 out of 35 subjects (5.7%)**  1) I think dia macam is accurate placement in my mouth right? (Subject 23, 21 years old Malay male wearing upper and lower VFR)  2) I think the whole quality is quite good, for now it’s still in good condition. Because the VFR doesn't have any bubbles, I think for me it is quite good. I think robustness is about quality or condition of the retainer, I think also for me is the accuracy of the placement. (Subject 18, 25 years old Chinese female wearing upper and lower VFR) | [Modified]  Changed to Strength and durability of retainer |
|  | 9. Understood it as stability | **1 out of 35 subjects (2.9%)**  1) When I wear it is it stable or easy to move around? (Subject 11, 25 years old Chinese female wearing upper and lower VFR) | [Modified]  Changed to Strength and durability of retainer |
|  | 10. Understood but found it confusing | **4 out of 35 subjects (11.4%)**  1) I am not sure what you mean , is it the retainer strong or what do you mean by it, I know it is strong but I am not sure in what term. (Subject 24, 38 years old Malay female wearing upper VFR)  2) Not easily spoiled, I did not have a container to keep it, I just put it anywhere, but it did not deformed, the shape is still there. I don't know how to describe robustness but I know it is about how strong my retainers are. (Subject 25, 21 years old Chinese female wearing upper and lower VFR)  3) Actually I am not really understand the robustness of the retainer. Is it about the strength of the retainer? (Subject 7, 20 years old Chinese female wearing upper and lower VFR)  4) Based on my own understanding is whether it is strong or not, but I was quite confused on the meaning. (Subject 26, 20 years old Chinese female wearing upper and lower VFR) | [Modified]  Changed to Strength and durability of retainer |
|  | 11. Found the meaning on google when answering | **3 out of 35 subjects (8.6%)**  1) Just now when I see the word, I don't really know what is that, then I just go and google search, then it says it is strength, I haven come across this word, so I google search. (Subject 27, 21 years old Chinese male wearing upper and lower VFR)  2) Only robustness is confusing cuz I googled it, I only have the idea but I just checked through then I was right. (Subject 28, 20 years old Indian female wearing upper and lower VFR)  3) Just now I checked on google, robustness means quality right? At first I didn't know the meaning. (Subject 20, 24 years old Malay male wearing upper and lower VFR) | [Modified]  Changed to Strength and durability of retainer |
|  | 12. Suggest to change the term “robustness” | **5 out of 35 subjects (14.3%)**  1) Maybe because I am a dental student, if for normal people, they won't understand what is robustness. (Subject 8, 22 years old Malay female wearing upper Hawley retainer and lower bonded retainer)  2) I think you should change it to something yang related sikit to the ‘strength’ (after the interviewer explained the term robustness) (Subject 23, 21 years old Malay male wearing upper and lower VFR)  3) Maybe just write the strength of the retainer, so the second I look at it I can understand. (Subject 27, 21 years old Chinese male wearing upper and lower VFR)  4) I think durability is more suitable. (Subject 29, 23 years old Malay female wearing upper and lower VFR)  5) I had to guess what it meant. Maybe there is a simpler word that you can use, maybe it will be better. (Subject 24, 38 years old Malay female wearing upper VFR) | [Reviewed]  Changed to Strength and durability of retainer |
| To put the description below each question |  | **8 out of 35 subjects (22.9%)**  1) It is good that it is simple, but sometimes too simple can be misleading. Instead of just putting the criteria, maybe you can put a little description below it. (Subject 9, 24 years old Malay male wearing upper and lower VFR)  2) Maybe mouth cleanliness can add on a bit of clarification, because mouth cleanliness brings me to retainer cleanliness. (Subject 2, 23 years old Chinese female wearing upper and lower VFR)  3) Maybe put small words, mouth cleanliness means mouth not retainer, just explain it, more specific. (Subject 10, 19 years old Malay male wearing upper and lower VFR)  4) Maybe the robustness can write the meaning below, what do you mean by robustness of retainer. (Subject 5, 20 years old Chinese female wearing upper and lower VFR)  5) I think you can just specifically mention what you want others to define about the robustness, it would be better. (Subject 18, 25 years old Chinese female wearing upper and lower VFR)  6) Boleh explain sikit perkataan 4 dan 5 tu. (Subject 13, 26 years old Malay female wearing upper and lower VFR and bonded retainer)  7) Can make the sentence longer? (Subject 7, 20 years old Chinese female wearing upper and lower VFR)  8) Maybe you can explain more on what you want to know when it comes to robustness and also regarding cleanliness. (Subject 14, 23 years old Iban female wearing upper and lower Hawley retainers) | [Reviewed]  No description added. |
